# Assessing changes in the quality of quantitative health educations research: a perspective from communities of practice

**DOI:** 10.1186/s12909-022-03301-1

**Published:** 2022-04-01

**Authors:** Katherine M. Wright, Larry D. Gruppen, Kevin W. Kuo, Andrew Muzyk, Jeffry Nahmias, Darcy A. Reed, Gurjit Sandhu, Anita V. Shelgikar, Jennifer N. Stojan, Toshiko L. Uchida, Rebecca Wallihan, Larry Hurtubise

**Affiliations:** 1 Department of Family & Community Medicine, Northwestern University Feinberg School of Medicine, Chicago, IL USA; 2 Department of Learning Health Sciences, University of Michigan, Ann Arbor, MI USA; 3 Stanford University, Palo Alto, CA USA; 4 Department of Medical Education, Duke University, Durham, NC USA; 5 University of California, Irvine, Orange, CA USA; 6 Mayo Clinic College of Medicine and Science, Rochester, MN USA; 7 Department of Surgery, University of Michigan, Ann Arbor, MI USA; 8 Department of Neurology, University of Michigan, Ann Arbor, MI USA; 9 Departments of Internal Medicine and Pediatrics, University of Michigan, Ann Arbor, MI USA; 10 Northwestern University Feinberg School of Medicine, Chicago, IL USA; 11 General Pediatrics Residency, Nationwide Children’s Hospital & The Ohio State College of Medicine, Columbus, OH USA; 12 The Michael V. Drake Institute for Teaching and Learning, The Ohio State University, Columbus, OH USA

**Keywords:** Medical education research, Research quality, Research methods, Methodological quality

## Abstract

**Background:**

As a community of practice (CoP), medical education depends on its research literature to communicate new knowledge, examine alternative perspectives, and share methodological innovations. As a key route of communication, the medical education CoP must be concerned about the rigor and validity of its research literature, but prior studies have suggested the need to improve medical education research quality. Of concern in the present study is the question of how responsive the medical education research literature is to changes in the CoP. We examine the nature and extent of changes in the quality of medical education research over a decade, using a widely cited study of research quality in the medical education research literature as a benchmark to compare more recent quality indicators.

**Methods:**

A bibliometric analysis was conducted to examine the methodologic quality of quantitative medical education research studies published in 13 selected journals from September 2013 to December 2014. Quality scores were calculated for 482 medical education studies using a 10-item Medical Education Research Study Quality Instrument (MERSQI) that has demonstrated strong validity evidence. These data were compared with data from the original study for the same journals in the period September 2002 to December 2003. Eleven investigators representing 6 academic medical centers reviewed and scored the research studies that met inclusion and exclusion criteria. Primary outcome measures include MERSQI quality indicators for 6 domains: study design, sampling, type of data, validity, data analysis, and outcomes.

**Results:**

There were statistically significant improvements in four sub-domain measures: study design, type of data, validity and outcomes. There were no changes in sampling quality or the appropriateness of data analysis methods. There was a small but significant increase in the use of patient outcomes in these studies.

**Conclusions:**

Overall, we judge this as equivocal evidence for the responsiveness of the research literature to changes in the medical education CoP. This study identified areas of strength as well as opportunities for continued development of medical education research.

**Supplementary Information:**

The online version contains supplementary material available at 10.1186/s12909-022-03301-1.

Health professions education is a landscape of practice made up of multiple Communities of Practice (CoP) [1–4]. CoP are groups of people who share a concern, a set of problems, or a passion about a topic, and who deepen their knowledge and expertise in this area by interacting on an ongoing basis. Barab, Barnett, and Squire stress that CoPs are persistent and develop mutual professional values and shared history [5].

Published research literature is clearly a critical component of an academic CoP. The scholarly literature reflects the three components of a CoP [2]. First, the published literature reflects the domain of a CoP. The domain is the common ground of relevant problems, topics of interest, knowledge, and practice that define the contributions and participation of members of the community. The domain has boundaries that help define the community as well as ‘leading edges’ for expanding or redirecting the domain.

Secondly, the literature reflects the community and social fabric of the CoP. As a vehicle for communication, the literature enables shared ideas, knowledge, and priorities. It also reflects the social networks within the community through collaborations and citations [6]. Thirdly, scholarly publications serve as a repository and resource of community practice. The literature is particularly important for identifying new techniques and methods, theoretical perspectives, findings, and language for the community.

CoPs change over time as new members enter into the core of the community and older members leave. They change as the domain of the community shifts and grows or shrinks (becomes more specialized). Changes in practice also changes the CoP. Many changes in the medical education CoP can be identified: the recent emphasis on competency-based education [7, 8], newer models of faculty development [9], the comings and goings of different curricular models (systems-based, problem-based, team-based), the shift in a predominantly male community in the 1970s to an increasingly gender diverse community in the early twenty-first century, and the movement from a preponderance of quantitative research methods to a breadth of quantitative, qualitative, and mixed methods.

Although change is inevitable in a CoP and the associated scholarly literature that is part of it, we know little about the dynamics of those changes. Of particular interest in the present paper is how and how quickly the characteristics of the scholarly literature change over time. Changes in the scholarly literature may be both the result of change as well as the agent of change in the CoP. Understanding the dynamics of change in the research literature informs appropriate selection and design of interventions to improve that communication stream within the medical education CoP.

Our research question for this study is “How much and what kinds of change take place in the quality of research literature for medical education over a (11 year) period of time?” The question of change in a CoP can be challenging. One must identify a specific outcome to evaluate over some period of time but neither outcome nor time period are obvious. Gathering outcomes data over a period of time is also difficult, given the paucity of databases that preserve these kinds of data. Literature databases (e.g., MEDLINE) often serve as the data source for such studies, either through an analysis of outcomes that can be assessed over a period of time, such as the academic disciplines represented in research topics [10], or a longitudinal examination of specific topics or themes, like clinical reasoning [11].

Another methodological approach is to identify an historic study and seek to replicate it sometime later. By comparing results before and after some intervening period, investigators can make observations about changes and their potential implications. One example of this approach examined eight units of medical education research, comparing individual reports in a special issue of Academic Medicine with new interviews of the original unit directors 14 years later [12]. The investigators analyzed transitions in community characteristics such as research productivity, community membership, and goals of the community.

For the present study, we have elected to follow a similar method to this last example. We identified a major study by Reed et al. [13], which examined the methodological strengths and weaknesses of the concurrent medical education literature by analyzing studies published in 13 medical and medical education journals between September 2002 and December 2003.

Since this initial work, there has been continued growth in the numbers of medical education research journals and conferences, the number of advanced degree programs in medical education scholarship) [14], as well as the number of individuals engaged in medical education research. Regulatory agencies increasingly mandate more rigor in educational assessment and innovation [15, 16], and the research and publication environment has become more competitive. However, it is unknown how medical education research quality has changed in tandem with these changes in the CoP.

We sought to investigate the nature and magnitude of potential changes in medical education research quality by replicating Reed, et al.’s study 11 years after the original analysis. We explored the question of whether the quality of medical education research studies would have increased, decreased or remained constant when reassessed after a period of time, using the same measures of study quality and the same journals to gauge how changes in the scholarly literature may relate to evolution of the medical education CoP.

## Methods

### Literature search and retrieval

An informationist with expertise in conducting literature searches guided the development of the search strategy with the goal of replicating Reed, et al. [13] using the same 13 peer-reviewed journals included in the initial study. These journals represent broad multidisciplinary medical research (JAMA, New England Journal of Medicine), seven core medical specialties (Academic Emergency Medicine, American Journal of Obstetrics and Gynecology, American Journal of Surgery, Annals of Internal Medicine, Family Medicine, Journal of General Internal Medicine, Pediatrics), as well as medical education-specific journals (Academic Medicine, Medical Education, Medical Teacher, Teaching and Learning in Medicine). The search was conducted on MEDLINE for research studies published from 9/01/2013 to 12/31/2014 to match the timeframe of the original study and included the keywords medical education and medical education research; MeSH term: Education, Medical (see appendix for full search syntax). The interval between the first and subsequent sample of the literature (11 years) reflects the time period in which the authors established their collaboration and began the time-consuming work of literature screening and abstraction and then data analysis, writing and publication. While this is not intended primarily as an indicator of current literature quality, it does provide insight into the evolution of communities of practice in medical education.

### Eligibility screening

Consistent with the previous study, medical education research was operationally defined as “any original research study pertaining to medical students, residents, fellows, faculty development, or continuing medical education for physicians” [13]. Studies focusing on patient education and/or non-physician clinicians were excluded. As in the original study, additional exclusion criteria were: qualitative studies (because the MERSQI does not assess the quality indicators of qualitative studies), meta-analyses and systematic reviews, clinical reviews, letters, editorials, and reports of educational interventions without any evaluation or outcomes.

Eleven of the authors participated in the screening and review process. As an initial calibration exercise, the research team reviewed articles outside the review sample for inclusion-exclusion decision agreement. Each of the 9286 articles in the review sample was then screened by arbitrary pairs of reviewers for inclusion-exclusion decisions. Disagreements between raters were arbitrated through group discussion until consensus was achieved. A kappa coefficient was calculated to estimate rater agreement in selection screening using a sub-sample of 10% (928 papers) and demonstrated moderate agreement between raters (Cohen kappa = 0.43).

After the title and abstract screening, the full-text of all articles meeting inclusion criteria were retrieved. The same inclusion and exclusion criteria as the title/abstract screen were then applied to these full-text articles. The full-text articles that met inclusion-exclusion criteria were abstracted for the study variables.

### Data abstraction

We used the Medical Education Research Study Quality Instrument (MERSQI) [13] to measure the methodological quality of medical education research studies. The MERSQI was designed to measure methodologic quality rather than the quality of reporting (but it is still dependent on the information provided in the written manuscript [17]). This instrument includes 10 items grouped into 6 domains of study quality including: study design (with options of single group cross-sectional or single group post-test only; single group pre and post-test; non-randomized, 2 group; and randomized controlled experiment), sampling (number of institutions (1, 2, or more) and response rate (< 50%, 50–74%; ≥ 75%), type of data (assessment by study subject; or objective measurement), validity evidence (internal structure, content, and relationships to other variables), data analysis (appropriateness and complexity), and outcomes (satisfaction, attitudes, perceptions, opinions, general facts; knowledge, skills; behaviors; patient/health care outcome). Each MERSQI domain has a maximum possible score of 3. Prior work documents an intraclass correlation coefficient for interrater reliability ranging from 0.72 to 0.98 for scoring the 6 domains [13].

The MERSQI has excellent inter- and intra-rater reliability in addition to strong validity evidence related to construct, content, and internal structure. The original MERSQI report has been widely cited in the medical education literature (86 citations in PubMed as of 19 January 2021). It is frequently used as a quality measure in systematic and other reviews in a wide range of medical fields [18–20] Validity evidence for assessing methodological and research characteristics has been reported [17, 21].

### Analyses

Descriptive statistics were calculated to explore indicators of study quality. Current data were compared to the Reed, et al., [13] results using chi-square tests for relative frequency data and t-tests for comparison of mean scores. The primary outcomes were the six mean MERSQI scores for the individual categories of study quality. These were calculated by standardizing the percentage of total achievable points after accounting for “not applicable” responses. A total score was not computed for the MERSQI, following recommendations of the original authors [17] .For all analyses, a two-tailed alpha level of 0.05 was used to determine statistical significance. Effect sizes are reported for all comparisons; Cohen’s d for t-tests and h for tests of two proportions [22] (Table 1). Data were analyzed using SPSS version 24 for Mac (IBM Corp., Armonk, New York) and R version 3.4.3 for Mac (R Foundation for Statistical Computing, Vienna, Austria).

**Table 1:** MERSQI domain and item scores for two cohorts of medical education research studies (published between 2002 and 2003 and 2013–2014)

				Original Study Manuscripts (***n*** = 210)			Replication Study Manuscripts (***n*** = 482)		
**Domain**	**Item**	**Item Score**	**Maximum Domain Score**	**Domain Score mean (SD)**	**Studies % (N)**		**Domain Score mean (SD)**	**Studies % (N)**	***P*** **value**^**b**^
Study Design			3	1.28 (0.47)			1.41 (0.66)		0.01^**b**^
	1. Study Design								
	Single group cross-sectional or single group post-test only	1			66.7 (140)			64.1 (309)	
	Single group pre and post-test	1.5			15.7 (33)			11.4 (55)	
	Non-randomized, 2 group	2			14.8 (31)			13.5 (65)	
	Randomized controlled experiment	3			2.9 (6)			11.0 (53)	
Sampling			3	1.90 (0.65)			1.92 (0.61)		0.71
	2. Institutions								
	Single institution	0.5			64.3 (135)			62.2 (300)	
	Two institutions	1			3.8 (8)			3.5 (17)	
	More than 2 institutions	1.5			31.9 (67)			34.2 (165)	
	3. Response Rate								
	Not applicable				14.3 (30)			14.2 (68)	
	Response rate < 50% or not reported	0.5			33.3 (60/180) ^a^			34.7 (143/414)^a^	
	Response rate 50–74%	1			21.7 (39/180) ^a^			21.4 (88/414)^a^	
	Response rate ≥ 75%	1.5			45.0 (81/180) ^a^			44.0 (181/414)^a^	
Type of Data			3	1.91 (0.99)			2.09 (.99)		0.03^**b**^
	4. Type of Data								
	Assessment by study subject	1			54.3 (114)			45.6 (220)	
	Objective measurement	3			45.7 (96)			54.4 (262)	
Validity of Evaluation Instruments’ Scores			3	0.69 (0.93)			1.06 (1.07)		< 0.001^**b**^
	5. Internal Structure								
	Not applicable				11.9 (25)			5.4 (26)	
	Not reported	0			74.6 (138/185) ^a^			71.1 (324/456)^a^	
	Reported	1			25.4 (47/185) ^a^			28.9 (132/456)^a^	
	6. Content								
	Not applicable				11.9 (25)			5.4 (26)	
	Not reported	0			71.4 (132/185)^a^			63.4 (289/456)^a^	
	Reported	1			28.6 (53/185)^a^			36.6 (167/456)^a^	
	7. Relationships to other variables								
	Not applicable				11.9 (25)			5.4 (26)	
	Not reported	0			84.9 (157/185)^a^			59.4 (271/456)^a^	
	Reported	1			15.1 (28/185)^a^			40.6 (185/456)^a^	
Data Analysis			3	2.58 (0.65)			2.64 (0.57)		0.22
	8. Appropriateness of analysis								
	Data analysis inappropriate for study design or type of data	0			13.8 (29)			6.6 (32)	
	Data analysis appropriate for study design and type of data	1			86.2 (181)			93.4 (450)	
	9. Sophistication of analysis								
	Descriptive analysis only	1			27.6 (58)			29.9 (144)	
	Beyond descriptive analysis	2			72.4 (152)			70.1 (338)	
Outcome			3	1.44 (0.50)			1.56 (0.61)		0.01^**b**^
	10. Outcome								
	Satisfaction, attitudes, perceptions, opinions, general facts	1			48.6 (102)			41.3 (199)	
	Knowledge, skills	1.5			19.5 (41)			24.1 (116)	
	Behaviors	2			29.5 (62)			25.5 (123)	
	Patient/health care outcome	3			2.4 (5)			9.1 (44)	
**Total**			18	9.95 (2.34)			10.71 (2.62)		< 0.001^**b**^

^a^ Percentage based on studies without a “not applicable” rating

^**b**^ Statistically significant at the .05 level

The Northwestern University Institutional Review Board deemed this study exempt from review (STU00205046).

## Results

### Identification of studies

**Fig. 1:**
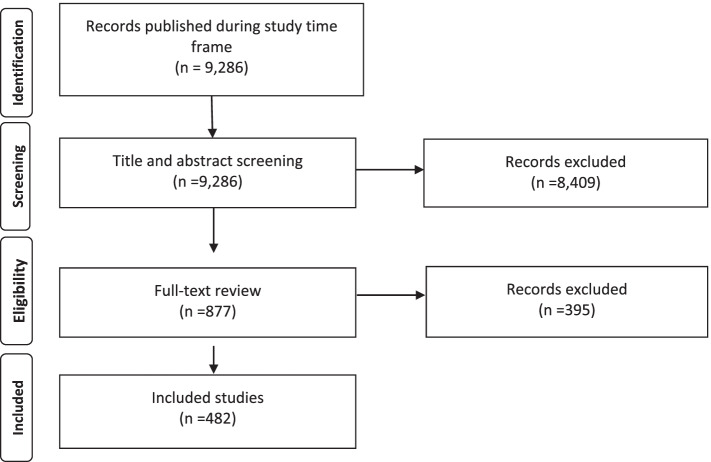
PRISMA flow diagram [23] showing the process for identifying and screening articles for inclusion in the study. Data were obtained from Ovid MEDLINE and included citation data for research studies published in 13 medical education journals between 2013 and 2014

A total of 9286 articles were initially identified by the search. After inclusion and exclusion screening, 877 (9.4%) articles remained. Full text articles were retrieved for these 877 articles and screened again, using the same inclusion and exclusion criteria. This resulted in 482 (55.0%) articles that went on to be coded for quality using the MERSQI tool. A summary of the eligibility screening process is presented in Fig. 1. Overall, 482 (5.2%) articles met eligibility criteria from the 2013–14 sample, compared to 210 articles (2.5%) out of 8505 total publications in the original Reed et al. study from 2002 to 03.

### Comparisons of study quality measures between 2002 and 03 vs. 2013–14

Consistent with the prior study, the highest mean domain quality score in the replication review was for the data analysis domain (mean = 2.6, SD 2.6, Table 1). The overall MERSQI score increased from 9.9 (SD 2.3) to 10.7 (SD 2.6) between 2002 and 03 and 2013–14 (*p* < 0.001). Of the six domains of study quality measured by the MERSQI, there were statistically significant improvements in four measures: study design, type of data, validity and outcomes. Scores that did not change significantly in the time between the two analyses were in the domains of data analysis and sampling.

The mean score on the study design domain improved from 1.3 to 1.4 (*p* < 0.01), but there were no statistically significant changes for any specific type of study design. The majority (64.1%) of designs continued to be single group cross-sectional or post-test only. Randomized control designs were still infrequent, although their relative proportion among published studies increased almost four-fold over this time period, from 2.9 to 11.0% of included studies.

For the sampling domain, the proportion of studies that were multi-institutional was stable over this period. Despite calls for more collaborative, multi-institutional research, there was little change over the intervening decade, with the majority of papers (62.2%) continuing to be single-institution studies.

The 2013–14 set of studies had a significantly greater use of objective measurements than the prior cohort of studies (45.7% of articles in 2002–03 and 54.4% of articles in 2013–14, *p* < 0.001).

The reporting of validity evidence for medical education research studies was a frequent deficiency in the literature from 2002 to 03 and, although there was a statistically significant improvement (from 0.69 in 2002–03 to 1.06 in 2013–14, *p* < 0.001), this was still the lowest scoring domain among all of the MERSQI dimensions (mean = 1.06 out of a possible maximum score of 3.00). The 2013–2014 analysis showed increased reporting of all three forms of validity evidence (internal structure, content, relationship to other variables) analyzed using the MERSQI, compared to the 2002–03 analysis.

The highest scores were in the domain of data analysis (2.6 and 2.6 out of 3.0, in 2002–03 and 2013–14, respectively), and these did not change significantly over the study time period (*p* = 0.22). The large majority of studies in both time periods were considered to have appropriate data analysis procedures for the data reported and most went beyond simple descriptive statistics to reflect the number and relationship among variables in the study.

Study outcome scores showed a small but statistically significant increase from the 2002–03 sample to the 2013–14 sample (means = 1.4 vs. 1.6, *p* = 0.01). The most common outcomes in both samples were attitudes, satisfaction, perceptions, opinions and general facts (48.6% of articles in 2002–03 and 41.3% of articles in 2013–14, *p* < .0.01)). Patient and health care outcomes were reported almost four times more frequently in the 2013–14 sample compared to the previous sample (2.4% of articles in 2002–03 and 9.1% of articles in 2013–14), yet these important outcomes were still reported in only a small fraction of medical education research studies.

## Discussion

The larger CoP for medical education research has changed over the past couple of decades in ways that these results may reflect. There has been an increase in the number of medical education research journals. This may have acted to decrease the number of submissions to the journals included in this study by spreading potential publications across a greater number of outlets. On the other hand, the percentage of articles meeting study inclusion criteria more than doubled from 2.5% in 2002–03 to 5.2% in 2013–14, which may indicate that these high-impact journals are attracting more high-quality submissions while less rigorous work has other outlets.

Similarly, the proliferation of professional societies and academic conferences related to medical education globally has grown significantly, which suggests that there are many more investigators producing research articles. This increased demand for journal space may have driven the increase in the number of journals, but the causal relationship is not clear.

These findings suggest that the methodological quality of quantitative medical education research improved from 2002 to 03 to 2013–14. This is encouraging, given the established need for increased methodological rigor, efforts to increase faculty skills in education research, and the recognized importance of a robust evidence base in medical education [24]. The improvement in methodologic quality reflects growth in both the domain of the CoP as well as the practice of medical education research itself.

Some of the most challenging components of study quality within medical education had notable gains between the two time periods. In particular, the inclusion of and attention to validity evidence for the measures used in the studies increased significantly from 2002 to 03 to 2013–14. The medical education research community has called for an emphasis on validity evidence for more than 20 years [25–28]. This, therefore, is a welcome improvement in medical education research quality as defined by the accuracy and relevance of the measurement methods used to acquire data. Reporting of patient and healthcare outcomes also increased nearly four-fold. Although only 9.1% of studies assessed patient outcomes in the 2013–14 cohort, this is an important step towards the ultimate goal of medical education—to improve health. At the same time, there was a comparable decrease in reliance on learner self-reported data such as satisfaction, opinions and self-assessments as primary outcome measures.

Our analysis also reveals that randomized controlled trials (RCTs) were being used more frequently in medical education in our analysis compared to the 2002–03, although RCTs still comprised only 11% of education studies. While RCTs are viewed as the gold standard in the clinical world, that is not necessarily the case in education. RCTs can be costly and time consuming to conduct and, in medical education, they may violate ethical principles related to withholding a potentially beneficial educational intervention from the learners who are randomized to the control arm. A well-designed quasi-experiment may generate more meaningful evidence than a poorly designed RCT. Methodological and ethical limitations unique to medical education warrant ongoing discussion around best practices in research design.

In 2013–14, nearly two thirds of education research studies still used single group designs. Single group studies are more convenient to conduct and often reflect the natural environment of education, which tends to provide curricular and teaching innovations for the entire learner group rather than segregate them into comparison conditions. Nonetheless, reliance on single-group designs hinders interpretation of the effects of the studied educational interventions.

Similarly, almost two-thirds of the studies in both samples were conducted at single institutions. This limits the generalizability of these studies to other settings, learners, and contexts. The lack of growth in multi-institutional studies over the period of this study is a concern and may partially reflect limited funding for medical education research. Indeed, in the 2002–03 cohort there was a much greater proportion of multi-institutional studies among studies with higher levels of funding, as multi-institutional collaboration facilitates rigorous, generalizable research but requires additional resources [29].

This study has several limitations. While the follow-up time period of 2013–14 is not current, the goal of this study was to examine the change in methodologic quality of medical education research and how the CoP is evolving, not to give a current snapshot of the medical education literature.

We also note that the MERSQI assesses aspects of study design, not study hypotheses or research questions. Study design needs to match the research question and single group, post study assessment may be a perfectly appropriate design for some research questions. In other words, our analyses implicitly assume that the content, focus, and questions are more or less consistent from the initial to the comparison time period. If that is not the case, changes in study design quality become more difficult to interpret. Another limitation of MERSQI is its lack of assessment of quality indicators of qualitative studies. The evolving interest in use of qualitative studies in medical education research demonstrates a shift in the CoP’s priorities, as qualitative studies have become foundational in medical education and other health professions education research.

Reviewers were not blind to the study authors or journals. We attempted to mitigate this issue by asking reviewers to recuse themselves from the review if a potential conflict of interest was noted. Additionally, inter-rater agreement on the screening decisions was only moderate (Cohen kappa = 0.43), which attenuates the ability to make statistically significant distinctions between our results and those of Reed et al. [13]. We acknowledge that our quality ratings were derived from published reports only, and publication requirements and practices (e.g., electronic appendices and other supplemental information) may limit the data that are included in publications, thereby impacting MERSQI scores. However, this was necessary to provide comparable data to Reed et al. [13].

In addition, in order to compare our data to Reed et al., our study focused solely on the journals that were included in the 2002–03 cohort. In contrast, an examination of all published education studies (across a wider array of journals) would provide useful data on the full body of medical education research. There has been a proliferation of journals that accept or are devoted to medical education research, but these new journals were excluded from this analysis to maintain consistency with the original study.

It is also very important to note that the original study and this replication only examined quantitative research. Any changes to the number and rigor of qualitative studies was not addressed in this study. To the extent that qualitative studies emphasize exploratory investigations and deeper understanding of mechanisms and phenomena, it may be that the inclusion of qualitative studies would increase the preponderance of outcomes in the attitudes, perceptions and opinions category over patient and health care outcomes.

Despite these limitations, our study may serve as a data point to chart the evolution of medical education research quality and its impact on the medical education research CoP. We found that quality improved from 2002 to 03 to 2013–14 as measured by the MERSQI. By 2013–14, a greater proportion of studies reported validity evidence and used patient-centered endpoints and more rigorous study designs. With continued attention to these areas, medical education research quality could continue to rise in coming years. Medical education research quality is positively associated with research funding [30] and this characteristic of the CoP may drive increases in resources dedicated to medical education resources. Engagement of the medical education research CoP with professional organizations, governmental and non-governmental groups may further support development of a high quality evidence base to guide medical education practice and further improve patient outcomes.

In terms of the larger question of how the research literature serves as a means of communication for the medical education CoP, these results may be a glass half full or half empty. Indeed, some characteristics of the literature show improvement over an 11-year period, yet others do not. The pace of change might also be disappointing to some who hope to see a more rapid transformation of the CoP toward an evidence base in education that supports adoption of new models of medical care, greater access to care, and a responsive educational system. Although the interpretation of these findings are open to discussion, we believe it does provide some encouragement for efforts to map the changes in the CoP with changes in one of its primary means of communicating information, values, and perspectives.

## Supplementary Information

**Additional file 1.**

## Data Availability

Data were obtained from Ovid MEDLINE.

## Abbreviations

CoP: Community of Practice
MERSQI: Medical Education Research Study Quality Instrument

## References

[1] Wenger EC, Snyder WM (2000). Communities of practice: the organizational frontier. Harv Bus Rev.

[2] Wenger E, McDermott RA, Snyder W (2002). Cultivating communities of practice: a guide to managing knowledge.

[3] van der Vleuten CPM (2014). Medical education research: a vibrant community of research and education practice. Med Educ.

[4] Wenger E, Trayner-Wenger B. Communities of practice: a brief introduction. :1–8. 2015. 10.2277/0521663636 Accessed 2018 Dec 8. http://wenger-trayner.com/wp-content/uploads/2015/04/07-Brief-introduction-to-communities-of-practice.pdf.

[5] Barab SA, Barnett M, Squire K (2002). Developing an empirical account of a community of practice: characterizing the essential tensions. J Learn Sci.

[6] Newman MEJ (2001). The structure of scientific collaboration networks. PNAS.

[7] Frank JR, Snell L, Englander R, Holmboe ES (2017). Implementing competency-based medical education: moving forward. Med Teach.

[8] Frank JR, Snell LS, Cate O ten, Holmboe ES, Carraccio C, Swing SR, Harris P, Glasgow NJ, Campbell C, Dath D, et al. 2010. Competency-based medical education: theory to practice. Med Teach 32(8):638–645. http://informahealthcare.com/doi/abs/10.3109/0142159X.2010.501190. 10.3109/0142159X.2010.501190 20662574

[9] Steinert Y (2000). Faculty development in the new millennium: key challenges and future directions. Med Teach.

[10] Norman G (2011). Fifty years of medical education research: waves of migration. Med Educ.

[11] Norman G (2005). Research in clinical reasoning: past history and current trends. Med Educ.

[12] Humphrey-Murto S, O’Brien B, Irby DM, van der Vleuten C, ten Cate O, Durning S, Gruppen L, Hamstra SJ, Hu W, Varpio L (2019). 14 years later: a follow-up case-study analysis of 8 health professions education scholarship units. Acad Med.

[13] Reed DA, Cook DA, Beckman TJ, Levine RB, Kern DE, Wright SM (2007). Association between funding and quality of published medical education research. JAMA.

[14] Tekian A, Harris I (2012). Preparing health professions education leaders worldwide: a description of masters-level programs. Med Teach.

[15] Swanson DB, Roberts TE (2016). Trends in national licensing examinations in medicine. Med Educ.

[16] Edgar L, Roberts S, Holmboe E. Milestones 2 . 0 : A Step Forward. J Grad Med Educ. 2018:367–9. 10.4300/JGME-D-18-00372.1 PMC6008021 29946411

[17] Cook DA, Reed DA (2015). Appraising the quality of medical education research methods: the medical education research study quality instrument and the Newcastle-Ottawa scale-education. Acad Med.

[18] Wasson LT, Cusmano A, Meli L, Louh I, Falzon L, Hampsey M, Young G, Shaffer J, Davidson KW (2016). Association between learning environment interventions and medical student well-being a systematic review. JAMA.

[19] Timberlake MD, Mayo HG, Scott L, Weis J, Gardner AK (2017). What do we know about intraoperative teaching?. Ann Surg.

[20] Geerts JM, Goodall AH, Agius S (2020). Evidence-based leadership development for physicians: a systematic literature review. Soc Sci Med.

[21] Sawatsky AP, Beckman TJ, Edakkanambeth Varayil J, Mandrekar JN, Reed DA, Wang AT (2015). Association between study quality and publication rates of medical education abstracts presented at the Society of General Internal Medicine annual meeting. J Gen Intern Med.

[22] Cohen J (1988). Statistical power analysis for the behavioral sciences.

[23] Moher D, Liberati A, Tetzlaff J, Altman DG, Altman D, Antes G, et al. Preferred reporting items for systematic reviews and meta-analyses: the PRISMA statement. PLoS Med. 2009;6(7). 10.1371/journal.pmed.1000097. 10.1371/journal.pmed.1000097 PMC2707599 19621072

[24] Gruppen LD, Yoder E, Frye A, Perkowski LC, Mavis B (2011). Supporting medical education research quality: the Association of American Medical Colleges’ medical education research certificate program. Acad Med.

[25] Chen FM, Burstin H, Huntington J (2005). The importance of clinical outcomes in medical education research. Med Educ.

[26] Kalet AL, Gillespie CC, Schwartz MD, Holmboe ES, Ark TK, Jay M, Paik S, Truncali A, Hyland Bruno J, Zabar SR (2010). New measures to establish the evidence base for medical education: identifying educationally sensitive patient outcomes. Acad Med.

[27] Chen C, Petterson S, Phillips R, Bazemore A, Mullan F (2014). Spending patterns in region of residency training and subsequent expenditures for care provided by practicing physicians for Medicare beneficiaries. JAMA.

[28] Chahine S, K Mahan K, Wright S, Monteiro S, LEM G, Barber C, Sebok-Syer SS, McConnell M, Yen W, De Champlain A (2018). A call to investigate the relationship between education and health outcomes using big data. Acad Med.

[29] Gruppen LD, Durning SJ (2016). Needles and haystacks: finding funding for medical education research. Acad Med.

[30] Reed DA, Kern DE, Levine RB, Wright SM (2005). Costs and funding for published medical education research. JAMA.

