# Supplementary material for: Assessing changes in the quality of quantitative health educations research: a perspective from communities of practice
Source: BMC Med Educ. 2022 Apr 1;22:227. doi: 10.1186/s12909-022-03301-1 (PMC8973642; doi:10.1186/s12909-022-03301-1)
Supplement: Supplementary file 1 — Additional file 1. [file 12909_2022_3301_MOESM1_ESM.docx]

**Appendix**

**Medline search syntax**

("JAMA"[Journal] OR "The New England journal of medicine"[Journal] OR "Academic medicine : journal of the Association of American Medical Colleges"[Journal] OR "Medical education"[Journal] OR "Teaching and learning in medicine"[Journal] OR "Medical teacher"[Journal] OR "Annals of internal medicine"[Journal] OR "Journal of general internal medicine"[Journal] OR "American journal of surgery"[Journal] OR "Pediatrics"[Journal] OR "Family medicine"[Journal] OR "American journal of obstetrics and gynecology"[Journal] OR "Academic emergency medicine : official journal of the Society for Academic Emergency Medicine"[Journal]) AND ("20130901"[Date - Publication] : "20141231"[Date - Publication]) AND (education, medical[MeSH Terms]) OR (medical education OR medical education research).
